# Case of a genodermatosis presenting with verrucous lesions mimicking treatment-refractory warts

**DOI:** 10.1016/j.jdcr.2026.01.036

**Published:** 2026-02-02

**Authors:** Raj P. Fadadu, Charisse Orme

**Affiliations:** Department of Dermatology, University of California, San Diego, San Diego, California

**Keywords:** cancer, Cowden syndrome, genetics, genodermatosis, hamartoma tumor syndrome, skin disease, warts

## Case description

A 52-year-old man presented to dermatology clinic with numerous, chronic verrucous lesions on the hands and feet. The lesions started on the hands and feet around 30 years ago and were becoming more numerous and spreading proximally. They were refractory to many topical and destructive wart therapies, including salicylic acid, 5-flourouracil, imiquimod, intralesional *Candida* antigen injections, cryotherapy, and shave removal. In addition, he had colon cancer at age 45 years, and his family history was notable for a brother and aunt with colon cancer.

**Fig 1:**
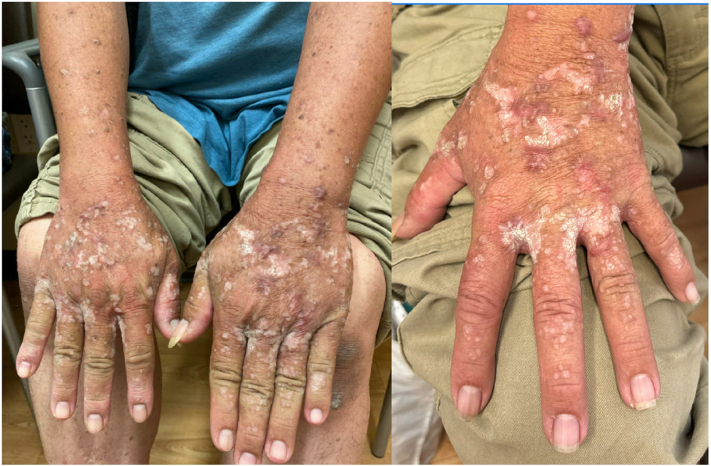
White verrucous papules and plaques and keloids on the hands and arms.

**Fig 2:**
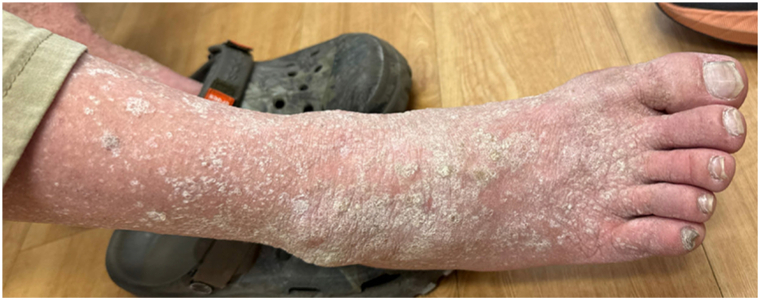
Confluent white and hyperkeratotic verrucous papules and plaques of the right foot and distal leg.

On examination, he had confluent verrucous papules and plaques on the bilateral hands and feet and distal arms and legs, with pink keloids on the hands (Figs 1 and 2). No intraoral pathologies were identified. Prior dermatopathology results from shave biopsies demonstrated verruca vulgaris, acral keratosis, and verrucous keratosis.

Labs initially ordered included complete blood cell count, complete metabolic panel, T-cell subset panel, HIV screening, immunoglobulin panel, and serum protein electrophoresis. Results were notable for CD8+ cell count of 149 (normal range: 180-1170 cells/μL), CD3+ cell count of 466 (840-3060 cells/μL), and CD4+ cell count of 303 (490-1740 cells/μL).

**Question 1: What is the most likely diagnosis?**

**A.** Warts, hypogammaglobulinemia, infections, and myelokathexis (WHIM) syndrome.
**B.** Epidermodysplasia verruciformis.
**C.** Multiple hamartoma tumor syndrome (Cowden syndrome).
**D.** Acquired natural killer-cell immunodeficiency.
**E.** Arsenic keratoses.

Correct answer: **C.** Multiple hamartoma tumor syndrome (Cowden syndrome).

## Discussion

After receiving the result of T-cell lymphopenia, we were concerned about immunodeficiency and genetic testing was ordered, which revealed a heterozygous pathogenic variant (c.1003C>T; p.Arg335∗) in the *PTEN* (phosphatase and tensin homolog) gene on chromosome 10, supporting a diagnosing of Cowden syndrome.

Cowden syndrome, or multiple hamartoma tumor syndrome, is a genodermatosis associated with mutations in the *PTEN* gene inherited in an autosomal dominant manner.^1^ It belongs to the *PTEN* hamartoma tumor syndrome spectrum, which also includes genodermatoses like Bannayan-Riley-Ruvalcaba syndrome. Physiologically, *PTEN* is a tumor suppressor gene that downregulates the phosphatidylinositol 3-kinase/AKT/mammalian target of rapamycin (mTOR) pathway.^1^ Loss of *PTEN* function leads to excessive mTOR signaling in certain tissues, causing high cellular proliferation. The gene also plays a role in immune development, though there are only few cases of Cowden syndrome presenting with immune dysregulation, such as T-cell lymphopenia in this patient.^2^

The mucocutaneous symptoms of Cowden syndrome encompass a broad spectrum, and most patients develop skin involvement early by age 30 years. The most common mucosal sites of involvement are the tongue and lips, which can develop cobblestone-appearing or papillomatosis lesions.^1^^,^^3^ Keratotic and verrucous papules on palmoplantar areas are often seen. Other skin findings include sclerotic fibromas, trichilemmomas of the central face, acrochordons, vascular malformations, and soft tissue tumors.^3^ Patients have a higher risk for melanoma (around 5% lifetime risk) compared to the general population. Regarding extra-mucocutaneous symptoms, there is increased risk for skeletal abnormalities, macrocephaly, neurocognitive delay, and numerous solid-organ malignancies, including colon, endometrial, breast, kidney, and thyroid.^4^

Management of mucocutaneous symptoms is primarily supportive, though there is limited evidence that topical or oral sirolimus, which inhibits mTOR, may be beneficial.^5^ Genetic counseling is important for patients and their families. Multidisciplinary management is essential for cancer surveillance given high risk for multiple solid-organ malignancies.

### Declaration of generative AI and AI-assisted technologies in the writing process

There was no use of artificial intelligence related to this article.

## Conflicts of interest

None disclosed.
